# Antioxidant Capacity-Related Preventive Effects of Shoumei (Slightly Fermented *Camellia sinensis*) Polyphenols against Hepatic Injury

**DOI:** 10.1155/2020/9329356

**Published:** 2020-08-19

**Authors:** Ruokun Yi, Yuxuan Wei, Fang Tan, Jianfei Mu, Xingyao Long, Yanni Pan, Weiwei Liu, Xin Zhao

**Affiliations:** ^1^Chongqing Collaborative Innovation Center for Functional Food, Chongqing University of Education, Chongqing 400067, China; ^2^Chongqing Engineering Research Center of Functional Food, Chongqing University of Education, Chongqing 400067, China; ^3^Chongqing Engineering Laboratory for Research and Development of Functional Food, Chongqing University of Education, Chongqing 400067, China; ^4^Second Clinical Medical College, Lanzhou University, Lanzhou, 730000 Gansu, China; ^5^Department of Public Health, Our Lady of Fatima University, Valenzuela 838, Philippines; ^6^Department of Food Science and Biotechnology, Cha University, Seongnam 13488, Republic of Korea; ^7^School of Public Health and Management, Chongqing Medical University, Chongqing 400016, China

## Abstract

Shoumei is a kind of white tea (slightly fermented *Camellia sinensis*) that is rich in polyphenols. In this study, polyphenols were extracted from Shoumei. High-performance liquid chromatography (HPLC) showed that the polyphenols included mainly gallic acid, catechin, hyperoside, and sulfuretin. In an *in vitro* experiment, H_2_O_2_ was used to induce oxidative damage in human normal hepatic L-02 cells. In an animal experiment, CCl_4_ was used to induce liver injury. The *in vitro* results showed that Shoumei polyphenols inhibited oxidative damage in normal hepatic L-02 cells, and the *in vivo* results showed that the polyphenols effectively reduced liver index values in mice with liver injury. The polyphenols also decreased aspartate aminotransferase (AST), alanine aminotransferase (ALT), alkaline phosphatase (ALP), triglyceride (TG), total cholesterol (TC), blood urea nitrogen (BUN), nitric oxide (NO), malondialdehyde (MDA), interleukin 6 (IL-6), interleukin 12 (IL-12), tumour necrosis factor alpha (TNF-*α*), and interferon gamma (IFN-*γ*) levels and increased albumin (ALB), superoxide dismutase (SOD), catalase (CAT), and glutathione peroxidase (GSH-Px) levels in the serum of mice with liver injury. Furthermore, pathological observation showed that the Shoumei polyphenols reduced CCl_4_-induced hepatocyte damage. qRT-PCR and Western blotting showed that the polyphenols upregulated the mRNA and protein expression of neuronal nitric oxide synthase (nNOS), endothelial nitric oxide synthase (eNOS), manganese- (Mn-) SOD, copper/zinc- (Cu/Zn-) SOD, CAT, and inhibitor of nuclear factor kappa B (NF-*κ*B) alpha (I*κ*B-*α*) and downregulated the expression of inducible nitric oxide synthase (iNOS) and NF-*κ*B p65. The Shoumei polyphenols had a preventive effect against CCl_4_-induced mouse liver injury equivalent to that of silymarin. The four polyphenols identified as the key substances responsible for this effect mediated the effect through their antioxidant capacity. These results suggest that Shoumei polyphenols are high-quality natural products with liver-protective effects.

## 1. Introduction

White tea (slightly fermented *Camellia sinensis*) is a commonly consumed tea in China. This tea is typically made from the shoot tips of tea plants. The colour of the finished tea leaves is light green with white frost, while that of the steeped tea is very light [1]. Shoumei is a white tea made from the buds and leaves of tea varietals that has been produced largely in Ningde City, Fujian Province, China [2]. Previous studies have shown that white tea exhibits various biological effects; for example, it reduces alcohol toxicity, lowers body temperature, moistens the lungs, improves blood circulation, relieves inflammation, enhances the detoxification system, decreases blood pressure and lipid levels, prevents fatigue, and calms and protects the liver [3–6].

The capability of polyphenolic extracts to scavenge hydrogen peroxide (H_2_O_2_), oxygen radicals, and hydroxyl radicals is concentration dependent, and the ability of these extracts to reduce and scavenge superoxide anions exceeds that of vitamin C [7]. In addition, the anti-inflammatory mechanisms by which plant polyphenols reduce oxidative stress involve primarily the antioxidant defence system and arachidonic acid metabolism. Under the influence of inflammatory factors, phagocytes can accumulate in inflammatory foci, release massive amounts of reactive oxygen species (ROS), and cause lipid peroxidation. However, the polyphenols in white tea can promote lysosomal release and reduce the secretion of various inflammatory factors [8].

Free radicals can lead to oxidative stress and eventually cause cancer; functional damage; chromosome changes; and cell, tissue, and organ ageing [9]. Carbon tetrachloride (CCl_4_) is a chemical intermediate that can be used to induce hepatic injury, and its principal mechanism is associated with both the chemical itself and its reactive metabolites. CCl_4_ can induce production of toxic metabolites in the liver via cytochrome P450 2El metabolic enzymes [10, 11]. ROS-induced oxidative stress can damage the liver to some extent, and such damage plays an important role in the progression of various types of hepatic injury [12].

Research has shown that white tea has antioxidant functions and enhances immunity *in vivo* [6]. In this study, the oxidative stress regulation-related effects of polyphenols from the white tea Shoumei on H_2_O_2_-induced oxidative damage in human normal hepatic L-02 cells and on CCl_4_-induced hepatic injury in mice were tested. In addition, specific Shoumei polyphenols were identified, and the mechanism of the effects of the polyphenols on CCl_4_-induced liver injury were investigated.

## 2. Materials and Methods

### 2.1. Extraction of Shoumei Polyphenols

After freeze drying, 1 kg of Shoumei tea (Fujian Guanglinfu Tea Co., Ltd., Ningde, Fujian, China) was weighed and ground into powder. Subsequently, the powdered tea was divided into 10 even parts. One litre of 70% (*v*/*v*) ethanol solution (Tianjin Damao Chemical Reagent Factory, Tianjin, China) was added to each part. The samples were leached in a 70°C water bath for 4 h and then subjected to filtration. The filtrates of the 10 samples were harvested and passed through diatomite to eliminate possible lipid-soluble impurities. All the extract solutions were repeatedly collected. The Shoumei tea polyphenols were adsorbed on resin via passage of the extraction solutions through an HP20 resin chromatography column (Mitsubishi Chemical Corporation, Tokyo, Japan). Then, the resin was eluted with 70% (*v*/*v*) ethanol, and the adsorbed polyphenols were dissolved in ethanol. Finally, the Shoumei polyphenol extract was evaporated with dry ethanol via rotary evaporation (R1020, Zhengzhou Instrument Factory, Zhengzhou, Henan, China) [13].

### 2.2. Analysis of Shoumei Polyphenols by High-Performance Liquid Chromatography (HPLC)

Ten milligrams of standard product (dried at 25°C for 24 h, Shanghai Yuanye Bio-Technology Co., Ltd., Shanghai, China) was accurately weighed to the nearest 0.01 mg. The standard was placed into a 10 mL volumetric flask, dissolved, and fixed with an appropriate amount of methanol. At the same time, 12.5 mg of polyphenol extract was accurately weighed, placed into a 25 mL volumetric flask, dissolved, and fixed with an appropriate amount of methanol. The flasks were shaken well to obtain 0.5 mg/mL test solutions. The polyphenolic components in the Shoumei tea samples were detected by chromatography under the following conditions using a diode array HPLC detector (Thermo Scientific Accucore C18 (2.6 *μ*m, 4.6 mm × 150 mm)): mobile phase A = 0.1%formic acid aqueous solution; mobile phase B = acetonitrile; column temperature = 30°C; flow rate = 0.5 mL/min; injection volume = 10 *μ*L; and detection wavelength = 359 nm (UltiMate 3000; Thermo Fisher Scientific, Waltham, MA, USA). The gradient elution conditions of the mobile phase are listed in Table 1.

### 2.3. Analysis of the Effects of Shoumei Polyphenols on Cell Survival Rates by MTT Assay

Human normal hepatic L-02 cells (Shanghai Institutes for Biological Science, Shanghai, China) in suspension (1 × 10^4^ cells/mL) were seeded into a 96-well cell culture plate (60 *μ*L of cell suspension + 100 *μ*L of medium) and cultured at 37°C for 24 h to adhere to the plate. Then, different concentrations of Shoumei polyphenol solution (0-200 *μ*g/mL; 20 *μ*L per well) were added, and the cells were incubated for 24 h. After that, 20 *μ*L of MTT (Thermo Fisher Scientific) was added, the cells were cultured for 4 h, the upper medium was removed, and 150 *μ*L of DMSO (Solarbio Life Sciences, Beijing, China) was added. The optical density (OD) value was measured at 494 nm after shaking for 30 min at 37°C in the dark. Three parallel wells were used for each group.

To prepare the oxidative damage model, L-02 cells were cultured using the abovementioned method. After the cells adhered to the plate, 20 *μ*L of H_2_O_2_ (0.3 mmol/L) was added, and the cells were cultured for 4 h. Different concentrations of Shoumei polyphenol solution (50, 100, and 200 *μ*g/mL; 20 *μ*L per well) were added, and the cells were cultured for another 24 h. After that, 20 *μ*L of MTT was added, the cells were cultured for 4 h, the upper medium was removed, and 150 *μ*L of DMSO was added. The OD values were measured.

### 2.4. Determination of Oxidase Activity In Vitro

After the cells were treated with the methods described in Section 2.3, the old culture medium was removed, and the cells were washed with precooled PBS. Then, 200 *μ*L of trypsin was added to detach the cells from the plate wall. The detached cells were transferred to a 1.5 mL centrifuge tube and centrifuged to remove the supernatant. The cells were again washed with precooled PBS and centrifuged at 4000 r/min for 15 min (BY-R10 Centrifuge, Beijing Baiyang Medical Equipment Co., Ltd., Beijing, China) to remove the supernatant. Normal saline (800 *μ*L) was added, and the cells were homogenized. The activity levels of the superoxide dismutase (SOD), glutathione peroxidase (GSH-Px), and catalase (CAT) enzymes in the cell homogenates were determined according to the instructions of the relevant kits (Nanjing Jiancheng Bioengineering Institute, Nanjing, Jiangsu, China).

### 2.5. Induction of Liver Injury

Fifty specific pathogen-free- (SPF-) grade Kunming mice (male, 6 weeks old, weighing 18-22 g) were obtained from Shanxi Medical University (Chongqing, China) and housed under a controlled room temperature of 25°C and a relative humidity of 60%. All mice were provided ad libitum access to drinking water and a chow diet. The animal bedding was replaced every 2 days. All mice were fed adaptively for 1 week and then assigned to 5 groups (*n* = 10 each): the model group (CCl_4_ group), the high-concentration Shoumei polyphenol (SPH) group, the low-concentration Shoumei polyphenol (SPL) group, the silymarin group, and the normal group. SPL and silymarin were diluted with distilled water to a concentration of 40 mg/mL and were gavaged according to the weights of the mice. The mice in the model and normal groups were administered normal saline by gavage; those in the SPH and SPL groups were treated with 200 and 100 mg/kg Shoumei polyphenols by gavage, respectively; and those in the silymarin group were treated with 200 mg/kg silymarin (Solarbio Life Sciences, Beijing, China) by gavage for 14 consecutive days. While the *in vitro* experiment was used to directly test cultured cells, the *in vivo* experiment involved administration through gastric perfusion. *In vivo*, the Shoumei polyphenols had to be absorbed by the mice in order to exert their effects, and some polyphenols were likely lost in the process; about 5% of the polyphenols could be metabolized in the blood. Thus, the concentrations used in this experiment were selected according to the methods in a previous study [1]. After 14 days of treatment, all mice were injected intraperitoneally with CCl_4_ (Tianjin Damao Chemical Reagent Factory, 0.1 mL/10 g; 1 : 1 CCl_4_ : olive oil (*v*/*v*)), except for those in the normal group [14]. Following CCl_4_ injection, all the treated mice were fasted for 24 h, and liver and blood samples were collected after sacrifice. The liver tissue index was calculated using the following equation: liver weight (g)/body weight of mouse (kg) × 100.

### 2.6. Determination of General Serum Levels

Blood samples were centrifuged at 4000 r/min for 10 min at 4°C, and the serum (upper layer of supernatant) was collected. The levels of albumin (ALB), alkaline phosphatase (ALP), alanine aminotransferase (ALT), aspartate aminotransferase (AST), blood urea nitrogen (BUN), CAT, GSH-Px, malondialdehyde (MDA), nitric oxide (NO), SOD, total cholesterol (TC), and triglyceride (TG) in mouse serum were detected with reagents according to the manufacturer's protocols (Nanjing Jiancheng Bioengineering Institute, Nanjing, Jiangsu, China). Ten-microlitre serum samples were added to the reagents in the different detection kits, the absorbance values were determined by spectrophotometry, and the concentrations were calculated.

### 2.7. Determination of Serum Cytokine Levels

Serum samples were obtained after centrifugation of blood samples at 4000 r/min for 10 min at 4°C. The serum levels of interleukin 6 (IL-6), interleukin 12 (IL-12), interferon gamma (IFN-*γ*), and tumour necrosis factor alpha (TNF-*α*) were detected according to the instructions of the ELISA kits (Abcam, Cambridge, MA, USA).

### 2.8. Pathological Evaluation

Hepatic tissue samples (0.5 cm^2^) were harvested from mice and fixed in 10% formalin solution for 2 days. Subsequently, the tissues were dehydrated, cleared, immersed in wax, embedded, and sectioned, and the sections were stained with haematoxylin and eosin (H&E). The morphological alterations in the tissue samples were monitored using an Olympus BX43 light microscope (Tokyo, Japan). The degree of hepatocellular damage was calculated as the percentage of the number of lobules destroyed and divided into none (%), mild (1-25%), moderate (26-50%), and severe (>50%).

### 2.9. Quantitative Real-Time PCR (qRT-PCR)

L-02 cells were treated with Shoumei polyphenols (50, 100, and 200 *μ*g/mL) using the methods described in Section 2.3. The L-02 cells and liver tissues from mice were ground using a biological sample homogenizer, TRIzol™ Reagent (Thermo Fisher Scientific) was used for RNA isolation, and the concentration of each RNA sample was adjusted to 1 *μ*g/*μ*L by dilution. For cDNA synthesis, the diluted RNA (1 *μ*L) was reverse-transcribed according to the manufacturer's protocol (Thermo Fisher Scientific). Then, 1 *μ*L of cDNA template was mixed with 10 *μ*L of SYBR Green PCR Master Mix (Thermo Fisher Scientific), 1 *μ*L of each forward and reverse primer (Table 2), and 7 *μ*L of sterile distilled water. qRT-PCR was run using the following conditions: 1 min at 95°C; 40 cycles of 15 s at 95°C, 30 s at 55°C, and 35 s at 72°C; 30 s at 95°C; and 35 s at 55°C. GAPDH was employed as the housekeeping gene. The relative expression levels of the target genes were determined by using the 2^−ΔΔ*Ct*^ method (StepOnePlus PCR instrument, Thermo Fisher Scientific) [15].

### 2.10. Hepatic Tissue Analysis by Western Blot Analysis

One hundred milligrams of total protein from hepatic tissue was homogenized with 1 mL of RIPA buffer and 10 *μ*L of PMSF (Thermo Fisher Scientific), lysed (4°C, 5 min), and centrifuged (12,000 r/min, 4°C, 15 min). The protein in the middle layer was quantified using a BCA kit (Easy Bio, Beijing, China). Each extracted protein sample was diluted to 50 *μ*g/mL, heated (100°C, 5 min) with sample buffer at a 4 : 1 ratio, and cooled on ice for 5 min. Acrylamide, resolving buffer, stacking buffer, distilled water, 10% ammonium persulphate (APS), and tetramethylethylenediamine (TEMED) (Whatman Schleicher & Schuell, Keene, NH, USA) were added and mixed to prepare SDS-PAGE running and stacking gels for loading. A prestained protein ladder and the samples were injected into the wells of the gels, and vertical SDS-PAGE was performed for 50 min. The PVDF membrane was activated with methanol for 1 min and then blocked with 1 × TBST (Solarbio Life Sciences, Beijing, China) containing 5% skimmed milk for 1 h. After blocking, the PVDF membrane was washed with 1 × TBST and incubated with primary antibodies (against neuronal NO synthase (nNOS), endothelial NO synthase (eNOS), inducible NO synthase (iNOS), CAT, copper/zinc- (Cu/Zn-) SOD, manganese- (Mn-) SOD, inhibitor of nuclear factor kappa B (NF-*κ*B) alpha (I*κ*B-*α*), NF-*κ*B, and *β*-actin, Thermo Fisher Scientific) at 25°C for 2 h. The membrane was further incubated with secondary antibodies at 25°C for 1 h after being washed with 1 × TBST five times. Finally, the PVDF membrane was sprayed with a detection reagent and then placed in an imaging system (Tanon 6200 Luminous Imaging Workstation, Tanon Science and Technology Co., Ltd., Shanghai, China) for observation [15].

### 2.11. Statistical Analysis

Three parallel experiments were carried out for all serum and tissue samples, and the mean values were taken. Then, Statistical Package for the Social Sciences software (SAS Institute Inc., Cary, NC, USA) was used for data analysis. An array of 5 groups was used to analyse the differences between any two groups. One-way analysis of variance was used to assess the significant differences among more than two groups. *P* values less than 0.05 were deemed to indicate statistical significance.

## 3. Results

### 3.1. Composition Analysis of Shoumei Polyphenol Extract

**Figure 1:**
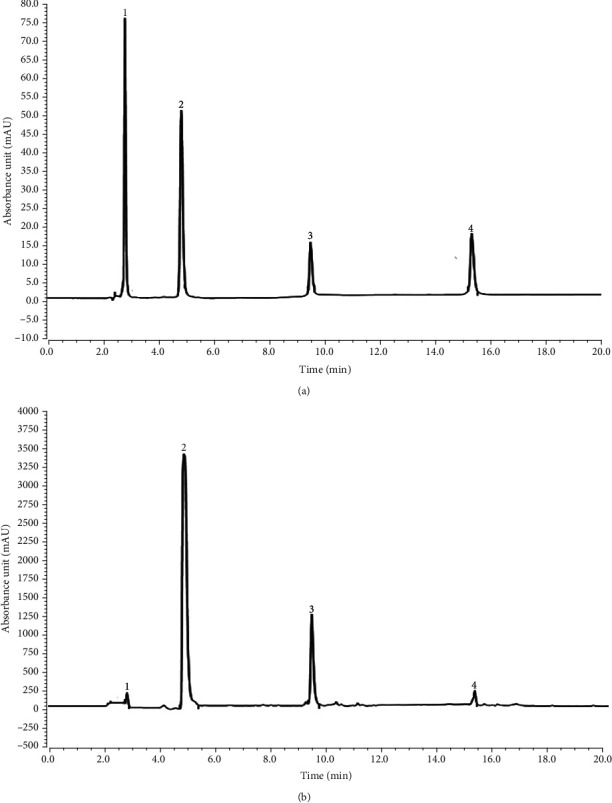
Polyphenol extract constituents of Shoumei. (a) Standard chromatograms. (b) Chromatograms of lotus leaf polyphenols. 1: Gallic acid; 2: catechin; 3: hyperoside; 4: sulfuretin.

Stock solutions of four standards (40 *μ*L each) were mixed evenly to obtain a mixed standard solution. For the liquid chromatography experiment, after the instrument was stable, the mixed standard was analysed. Then, the four individual standards (50 *μ*L of standard stock solution diluted 20-fold) were analysed under the same liquid chromatography conditions, and the results were compared with the chromatogram information for the test solution. Ten-milligram Shoumei samples were weighed precisely, and 1 mL of DMSO was added. The mixtures were heated for 30 min at 60°C in a water bath, and then 1.5 mL of pure water and 1.5 mL of liquid phase methanol were added. The mixtures were again heated for 10 min in a water bath at 60°C, centrifuged for 5 min at 2000 r/min, and finally passed through a 0.22 *μ*m organic filter membrane for standard determination. It was determined that there were four polyphenols in Shoumei: gallic acid, catechin, hyperoside, and sulfuretin (Figure 1). Then, the content of each polyphenol in the Shoumei extract was calculated according to the linear regression equation for the compound, and the relevant, detection limit, precision, and repeatability data are shown in Table 3.

### 3.2. Cell Growth

**Figure 2:**
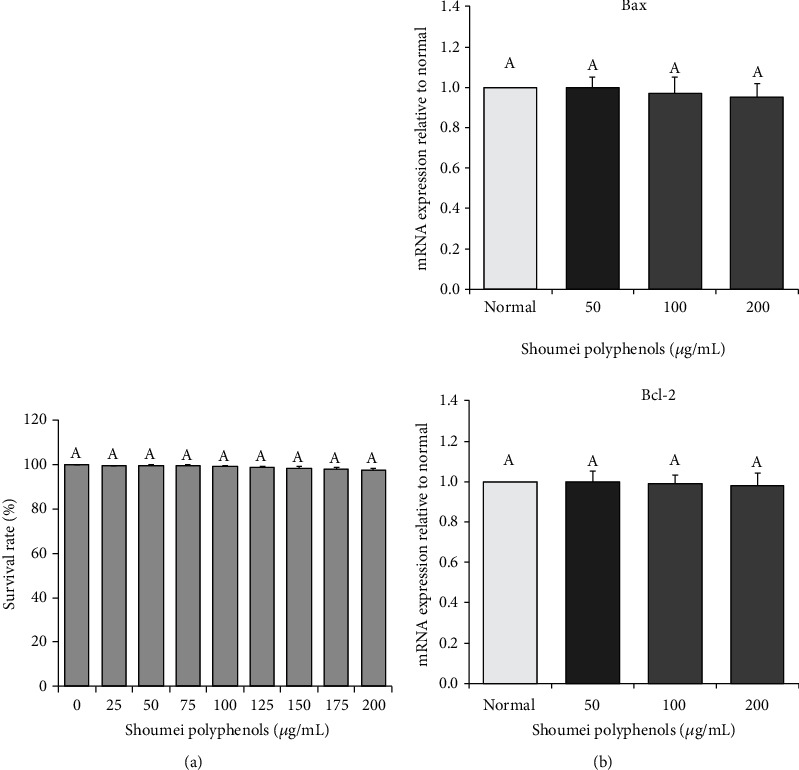
Effects of Shoumei polyphenols on survival rate of human normal hepatic L-02 cells. Values presented are the mean ± standard deviation (*N* = 10/group). Sample data in each group come from normal distribution, and the difference in variance between the two groups was significant (*P* > 0.05). ^a-e^Mean values with different letters over the bar are significantly different (*P* < 0.05) according to Tukey's honestly significant difference. Normal: untreated L-02 cells; oxidative damage: hydrogen peroxide-treated L-02 cells.

As shown in Figure 2(a), at concentrations of 0-200 *μ*g/mL, Shoumei polyphenols did not affect normal hepatic L-02 cell proliferation. In addition, the results of qRT-PCR showed that there was no difference in the expression of the apoptosis-related genes Bax and Bcl-2 between the Shoumei polyphenol-treated L-02 cells and the untreated L-02 cells (Figure 2(b)). The Shoumei polyphenols had no toxic or apoptosis-inducing effects on L-02 cells.

**Figure 3:**
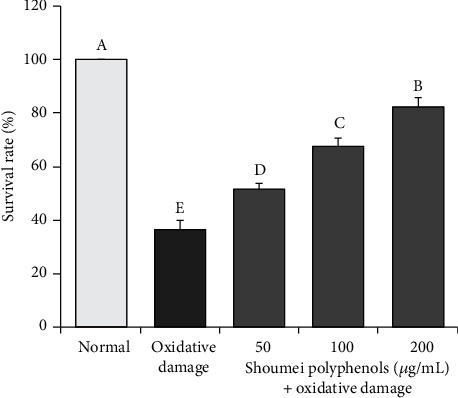
Effects of Shoumei polyphenols on survival rate of hydrogen peroxide-induced oxidative damage in human normal hepatic L-02 cells. Values presented are the mean ± standard deviation (*N* = 10/group). Sample data in each group come from normal distribution, and the difference in variance between the two groups was significant (*P* > 0.05). ^a-e^Mean values with different letters over the bar are significantly different (*P* < 0.05) according to Tukey's honestly significant difference. Normal: untreated L-02 cells; oxidative damage: hydrogen peroxide-treated L-02 cells.

As shown in Figure 3, the survival rate of normal hepatic L-02 cells damaged by H_2_O_2_ was significantly (*P* < 0.05) lower than that of normal cells. Compared with that of the non-polyphenol-treated H_2_O_2_-damaged cells, the survival rates of the H_2_O_2_-damaged cells treated with Shoumei polyphenol solution at 50, 100, and 200 *μ*g/mL were significantly (*P* < 0.05) higher. In addition, the protective effect of the high concentration (200 *μ*g/mL, 82.3%) was significantly greater than those of the lower concentrations (*P* < 0.05, 67.8% at 100 *μ*g/mL and 51.6% at 50 *μ*g/mL).

### 3.3. SOD, GSH-Px, and CAT Activity in Cells

**Figure 4:**
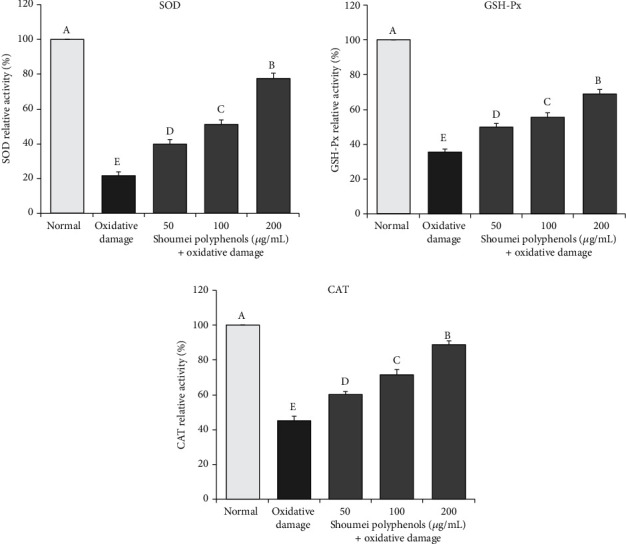
Effects of Shoumei polyphenols on SOD, GSH-Px, and CAT activities in hydrogen peroxide-induced oxidative damage in human normal hepatic L-02 cells. Values presented are the mean ± standard deviation (*N* = 10/group). Sample data in each group come from normal distribution, and the difference in variance between the two groups was significant (*P* > 0.05). ^a-e^Mean values with different letters over the bar are significantly different (*P* < 0.05) according to Tukey's honestly significant difference. Normal: untreated L-02 cells; oxidative damage: hydrogen peroxide-treated L-02 cells.

As shown in Figure 4, the levels of SOD, GSH-Px, and CAT activities in L-02 cells treated with H_2_O_2_ (0.3 mmol/L) for 4 h were significantly lower than those in normal cells. However, treatment with Shoumei polyphenols at different concentrations (50, 100, and 200 *μ*g/mL) significantly (*P* < 0.05) improved the activity levels of SOD (39.8%, 51.2%, and 77.4%), GSH-Px (49.8%, 55.6%, and 68.7%), and CAT (60.3%, 71.5%, and 88.7%) in H_2_O_2_-treated cells, and the improvement was greatest in the group treated with the high dose of Shoumei polyphenols.

### 3.4. mRNA Expression of I*κ*B-*α* and NF-*κ*B in L-02 Cells

**Figure 5:**
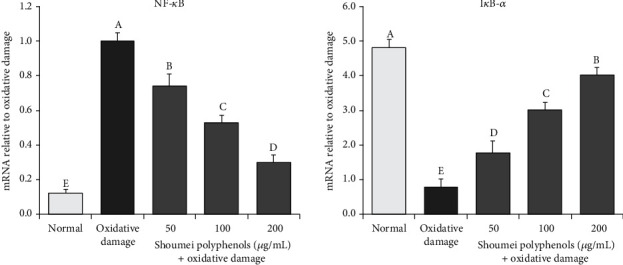
I*κ*B-*α* and NF-*κ*B mRNA expression in hydrogen peroxide-induced oxidative damage in human normal hepatic L-02 cells. Values presented are the mean ± standard deviation (*N* = 10/group). Sample data in each group come from normal distribution, and the difference in variance between the two groups was significant (*P* > 0.05). ^a-e^Mean values with different letters over the bar are significantly different (*P* < 0.05) according to Tukey's honestly significant difference. Normal: untreated L-02 cells; oxidative damage: hydrogen peroxide-treated L-02 cells.

As shown in Figure 5, the mRNA expression of NF-*κ*B was higher in cells with oxidative damage than in normal cells, while the expression of I*κ*B-*α* was lower in cells with oxidative damage. Shoumei polyphenol treatment decreased the expression of NF-*κ*B and increased that of I*κ*B-*α* in cells with oxidative damage, and the effects were stronger at higher concentrations of Shoumei polyphenols. The expression of NF-*κ*B and I*κ*B-*α* in the cells treated with Shoumei polyphenols at 200 *μ*g/mL (0.30- and 3.91-fold in the oxidative damage group) was the closest to that in normal cells.

### 3.5. Liver Index Values

As presented in Table 4, the mouse liver index values were highest in the model group and lowest in the normal group. Treatment with Shoumei polyphenols markedly reduced the liver index values in mice with CCl_4_-induced hepatic injury, and the efficacy of SPH was comparable to that of silymarin (1.09-fold in the silymarin group). The effects of SPH and silymarin were better than those of SPL (0.69-fold in the SPL group). Thus, the Shoumei polyphenols effectively reduced liver index values in mice with hepatic injury, and the effect was stronger at higher concentrations.

### 3.6. Serum Liver Function Indicators

As demonstrated in Table 5, the serum levels of ALT, AST, ALP, TG, TC, and BUN were lower in the normal group than in the other groups, while the serum level of ALB was higher in the normal group than in the other groups. The serum levels of ALT, AST, ALP, TG, TC, and BUN in the model group with CCl_4_-induced hepatic injury were higher than those in the other groups, but the ALB level was lower in the model group than in the other groups. SPH reduced the serum levels of ALT, AST, ALP, TG, TC, and BUN and increased those of ALB in mice with liver injury (model group); the ALT, AST, ALP, TG, TC, and BUN levels of the SPH group were significantly (*P* < 0.05) higher than the normal group, and the ALB level was significantly (*P* < 0.05) lower than the normal group. These effects were stronger than those of SPL (*P* < 0.05; 1.75-, 2.08-, 1.83-, 2.17-, 1.53-, 1.43-, and 0.73-fold in the SPH group) and similar to those of silymarin (0.89-, 0.95-, 0.92-, 0.88-, 0.95-, 0.94-, and 1.09-fold in the SPH group). SPL also reduced ALP, ALT, AST, BUN, TC, and TG levels and increased ALB levels compared to the levels in the model group, but its effects were weaker than those of SPH.

### 3.7. Serum Oxidation-Related Indicators

Table 6 shows that the serum levels of CAT, GSH-Px, and SOD in the normal group were markedly higher than those in the other four groups (*P* < 0.05), while the serum levels of MDA and NO in the normal group were markedly lower than those in the other four groups (*P* < 0.05). In contrast, the model group exhibited the lowest serum levels of CAT, GSH-Px, and SOD and the highest serum levels of MDA and NO. Shoumei polyphenol treatment reversed the CCl_4_-induced decreases in SOD, CAT, and GSH-Px levels and the CCl_4_-induced increases in NO and MDA levels. Notably, the effects of SPH were better than those of SPL (0.75-, 0.52-, 0.72-, 1.36-, and 1.86-fold in the SPH group) and comparable to those of silymarin (1.03-, 1.02-, 1.06-, 0.95-, and 0.97-fold in the SPH group).

### 3.8. Serum Cytokine Indicators

As presented in Table 7, the serum levels of IFN-*γ*, IL-6, IL-12, and TNF-*α* in the normal group were markedly lower than those in the other four groups (*P* < 0.05), while those in the model group were markedly higher than those in the other four groups (*P* < 0.05). Shoumei polyphenol treatment dramatically decreased the serum levels of the cytokines IL-6, IL-12, TNF-*α*, and IFN-*γ* in mice with hepatic injury. The efficacy of SPH was comparable to that of silymarin (0.95-, 0.95-, 0.95-, and 0.87-fold in the SPH group) and stronger than that of SPL (1.59-, 1.70-, 2.01-, and 1.42-fold in the SPH group).

### 3.9. Histopathological Observation of Hepatic Tissue

**Figure 6:**
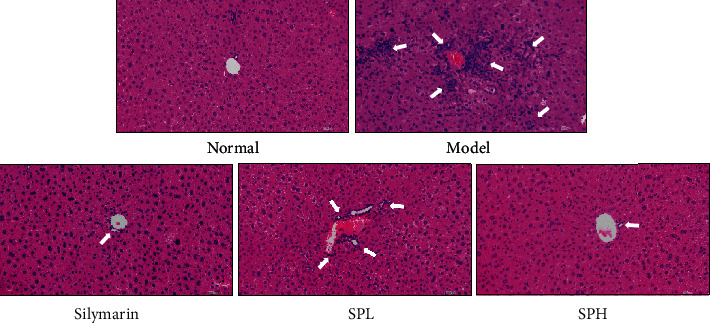
Histopathological observation of the liver of mice with CCl_4_-induced hepatic damage. Normal: untreated mice; model: CCl_4_-treated mice; SPL: 100 mg/kg Shoumei polyphenols and CCl_4_-treated mice; SPH: 200 mg/kg Shoumei polyphenols and CCl_4_-treated mice; silymarin: 200 mg/kg silymarin and CCl_4_-treated mice.

The structures of the liver tissues are illustrated in Figure 6. Notably, in the normal group, the hepatocytes were distributed radially around the central vein; in contrast, in the model group, the cells were unevenly arranged, the central vein was abnormal, the cellular structure was damaged, and the majority of cells exhibited necrosis. Morphological analysis revealed that Shoumei polyphenols and silymarin reduced necrosis of hepatocytes and ameliorated the morphological disruption of hepatic tissue induced by CCl_4_. The effect of SPH was superior to that of SPL and comparable to that of silymarin. The degree of damage of hepatic lobules in the model group reached 31.9%, and SPH could reduce the damage degree to 8.4%, which was stronger than SPL (20.8%; 2.48-fold in SPH), similar to silymarin (7.7%; 0.92-fold in SPH).

### 3.10. mRNA and Protein Expression of eNOS, iNOS, and nNOS in Hepatic Tissue

**Figure 7:**
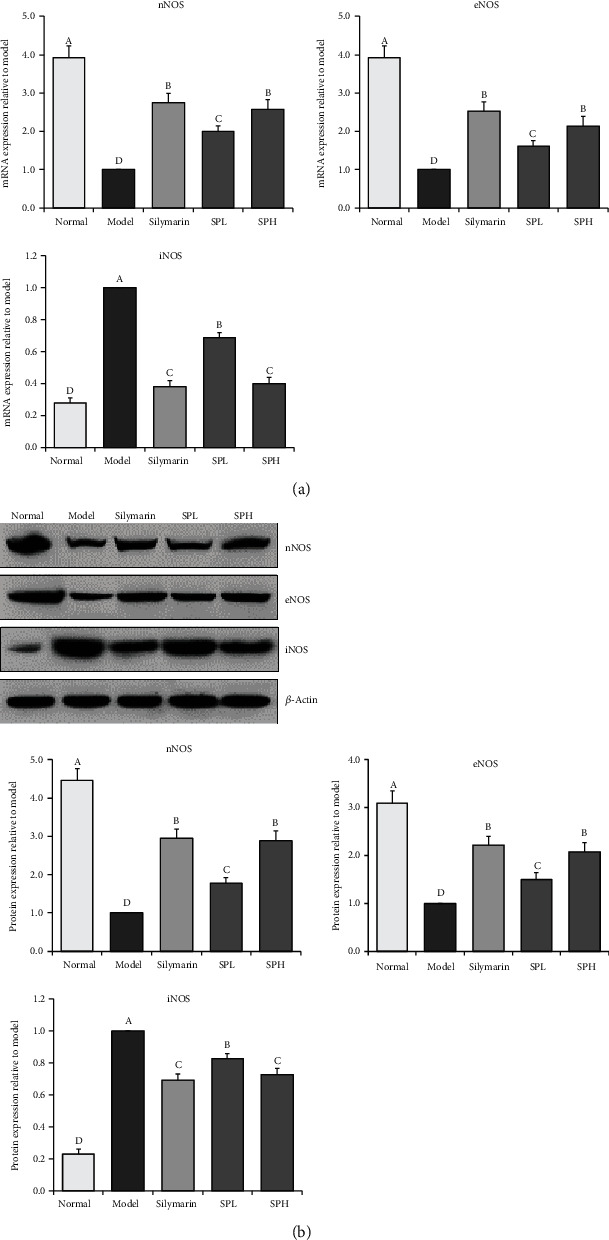
nNOS, eNOS, and iNOS mRNA (a) and protein (b) expression in liver tissue of mice with CCl_4_-induced hepatic damage. Values presented are the mean ± standard deviation (*N* = 10/group). Sample data in each group come from normal distribution, and the difference in variance between the two groups was significant (*P* > 0.05). ^a-e^Mean values with different letters over the bar are significantly different (*P* < 0.05) according to Tukey's honestly significant difference. Normal: untreated mice; model: CCl_4_-treated mice; SPL: 100 mg/kg Shoumei polyphenols and CCl_4_-treated mice; SPH: 200 mg/kg Shoumei polyphenols and CCl_4_-treated mice; silymarin: 200 mg/kg silymarin and CCl_4_-treated mice.

As demonstrated in Figure 7, the normal group exhibited the highest mRNA and protein expression of nNOS and eNOS and the lowest mRNA and protein expression of iNOS. Notably, CCl_4_ decreased the hepatic expression of eNOS and nNOS but increased that of iNOS. Shoumei polyphenol treatment downregulated the expression of iNOS and upregulated that of eNOS and nNOS. The effects of SPH were comparable to those of silymarin (0.98-, 1.04-, and 1.03-fold mRNA expression and 0.95-, 1.07-, and 1.03-fold protein expression of SPH). Both SPH and silymarin showed stronger effects than SPL (1.84-, 0.64-, and 0.74-fold mRNA expression and 1.14-, 0.72-, and 0.61-fold protein expression of SPH).

### 3.11. mRNA and Protein Expression of CAT, Gu/Zn-SOD, and Mn-SOD in Hepatic Tissue

**Figure 8:**
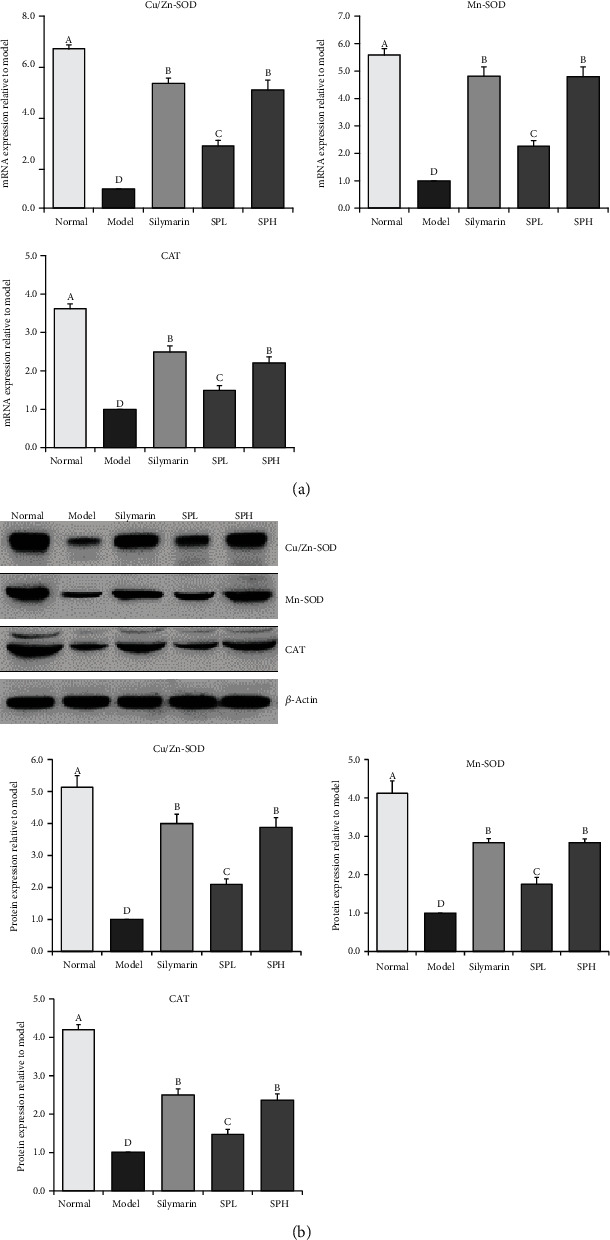
CAT, Gu/Zn-SOD, and Mn-SOD mRNA (a) and protein (b) expression in liver tissue of mice with CCl_4_-induced hepatic damage. Values presented are the mean ± standard deviation (*N* = 10/group). Sample data in each group come from normal distribution, and the difference in variance between the two groups was significant (*P* > 0.05). ^a-e^Mean values with different letters over the bar are significantly different (*P* < 0.05) according to Tukey's honestly significant difference. Normal: untreated mice; model: CCl_4_-treated mice; SPL: 100 mg/kg Shoumei polyphenols and CCl_4_-treated mice; SPH: 200 mg/kg Shoumei polyphenols and CCl_4_-treated mice; silymarin: 200 mg/kg silymarin and CCl_4_-treated mice.

As presented in Figure 8, the mRNA and protein expression levels of CAT, Gu/Zn-SOD, and Mn-SOD were highest in the normal group, while the levels of these genes were lowest in the model group. Silymarin significantly (*P* < 0.05) increased the hepatic expression of CAT, Gu/Zn-SOD, and Mn-SOD in mice with liver injury. Shoumei polyphenols also increased the expression levels of these genes, and the effects of SPH were more obvious than those of SPL (0.73-, 0.65-, and 0.48-fold mRNA expression and 0.62-, 0.53-, and 0.60-fold protein expression of SPH). There were no significant differences in the expression levels of these genes between the SPH and silymarin groups (*P* > 0.05; 1.11-, 1.14-, and 1.00-fold mRNA expression and 1.03-, 1.03-, and 1.01-fold protein expression of SPH).

### 3.12. mRNA and Protein Expression of I*κ*B-*α* and NF-*κ*B p65 in Hepatic Tissue

**Figure 9:**
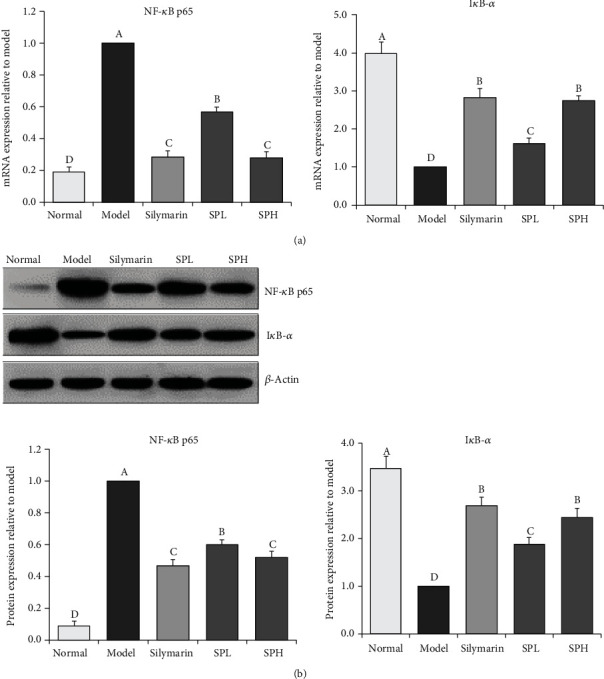
I*κ*B-*α* and NF-*κ*B mRNA (a) and protein (b) expression in liver tissue of mice with CCl_4_-induced hepatic damage. Values presented are the mean ± standard deviation (*N* = 10/group). Sample data in each group come from normal distribution, and the difference in variance between the two groups was significant (*P* > 0.05). ^a-e^Mean values with different letters over the bar are significantly different (*P* < 0.05) according to Tukey's honestly significant difference. Normal: untreated mice; model: CCl_4_-treated mice; SPL: 100 mg/kg Shoumei polyphenols and CCl_4_-treated mice; SPH: 200 mg/kg Shoumei polyphenols and CCl_4_-treated mice; silymarin: 200 mg/kg silymarin and CCl_4_-treated mice.

As shown in Figure 9, the expression of NF-*κ*B p65 in the model group with CCl_4_-induced hepatic injury was markedly downregulated compared to that in the other four groups (*P* < 0.05), whereas the expression of I*κ*B-*α* in the normal group was markedly reduced compared to that in the other four groups (*P* < 0.05). Indeed, the expression patterns of NF-*κ*B p65 and I*κ*B-*α* in the normal group were opposite to those in the model group. Our results indicated that the silymarin and Shoumei polyphenol treatment groups exhibited lower expression of NF-*κ*B p65 and higher expression of I*κ*B-*α* than the model group. In addition, the ability of SPH to downregulate NF-*κ*B p65 expression and upregulate I*κ*B-*α* expression was similar to that of silymarin (0.96- and 1.05-fold mRNA expression and 0.91- and 1.05-fold protein expression of SPH). NF-*κ*B p65 expression in SPL group mice (2.28-fold mRNA expression and 1.17-fold protein expression of SPH) was lower only than that in model group mice, while I*κ*B-*α* expression in SPL group mice (0.58-fold mRNA expression and 0.73-fold protein expression of SPH) was higher only than that in model group mice.

## 4. Discussion

Cell *in vitro* experiments can be used to preliminarily detect the biological activity of active substances. In this study, we examined the inhibitory effect of Shoumei polyphenols on H_2_O_2_-induced oxidative damage in cultured normal hepatocytes *in vitro* and found that Shoumei polyphenols effectively inhibited oxidative damage and protected hepatocytes. Moreover, Shoumei polyphenols did not impair the proliferation of normal hepatocytes or induce apoptosis.

The liver plays vital roles in maintaining homeostasis of body fluids and in regulating the metabolic rate. Although dietary patterns, lifestyle habits, and environmental conditions have improved, the incidence of liver disease is still increasing each year [16]. Silymarin, a traditional hepatoprotective drug, has long been applied for the treatment of various types of liver injury. It exhibits antioxidative, anti-inflammatory, immunoregulatory, and cell-regenerative effects [17]. Silymarin can reduce lipid peroxidation induced by CCl_4_ metabolites and by reduced coenzyme II (NADPH) alone, suggesting that silymarin is a chain-breaking antioxidant or a free radical scavenger. In the present work, silymarin was employed as a positive control drug. Both liver weight and the liver index were assessed to determine the degree of CCl_4_-induced liver injury. High liver weights and liver index values have been reported to be indicators of hepatic injury [18]. Similar findings were obtained in the present study. There are some differences in the mechanism of action of silymarin and Shoumei polyphenols on carbon tetrachloride-induced liver injury, but overall, both of them play a role by exerting their antioxidant capacity. The results showed that Shoumei polyphenols decreased liver weights and liver index values in mice with CCl_4_-induced liver injury with an efficacy comparable to that of silymarin.

AST is present predominantly in the mitochondria and cytoplasm of hepatocytes, whereas ALT is present predominantly in the cytoplasm of hepatocytes. Necrosis of hepatocytes can increase the levels of AST and ALT in the body [19]. In addition, lipid peroxidation induces changes in hepatocyte membrane permeability and causes hepatic ALP levels to increase sharply [20]. Hepatic injury can result in the transfer of fatty acids into the liver, leading to an elevated TG content in the liver. In addition, the degree of lipid peroxidation can be reflected by the hepatic levels of TG and TC [21]. Hepatic injury further damages kidney function and increases the content of BUN, a product of protein metabolism, in the bloodstream. Hepatic injury also impairs the synthesis, transport, and secretion of ALB in the body and reduces ALB content in the bloodstream [22]. In the present study, Shoumei polyphenols restored the above blood indexes to near-normal levels, and their effects were similar to the effects of silymarin.

CCl_4_ treatment can lead to excessive oxidative stress. The body can defend itself against oxidative damage via two pathways, namely, the enzymatic and nonenzymatic antioxidant pathways. Regulation by CAT, GSH-Px, and SOD may serve as the prominent mechanism of enzymatic antioxidant activity in the body [23]. CAT is a crucial antioxidant enzyme that eliminates H_2_O_2_, thereby reducing oxidative stress, inhibiting CCl_4_-induced oxidation and attenuating liver damage [24]. SOD catalyses the degradation of superoxide free radicals, which can lead to free radical removal. Synergistic activity between SOD and CAT has been found to enhance the clearance of free radicals [25]. GSH-Px is another important enzyme that catalyses the degeneration of H_2_O_2_. H_2_O_2_ reduction catalysed by GSH-Px can strengthen the cell membrane and prevent cell damage [26]. MDA is a metabolic product of lipid peroxidation that can accumulate in the body after hepatic injury [27]. Increases in the levels of NO and its oxidation products can disrupt cell-surface proteins and phospholipids, thus promoting inflammation and tissue injury [28]. In addition, NO can react with superoxide anions to form the peroxynitrite anion (ONOO^−^), which aggravates the oxidative stress reaction and further induces cytotoxicity and hepatic damage [29]. In this study, Shoumei polyphenols increased the serum levels of CAT, GSH-Px, and SOD and decreased the activity of MDA and NO in mice with hepatic injury.

CCl_4_ treatment can also lead to an inflammatory response in the liver in mice, as reflected by increased serum levels of IFN-*γ*, IL-6, IL-12, and TNF-*α* [30]. IL-6 is a factor released by Th2 cells that participates in humoral immunity. When IL-6 expression by Th2 cells increases, loss of visceral function can occur [31]. IL-6 can induce the proliferation and differentiation of T lymphocytes as well as the production of antibodies. It can also alter G cell activity, upregulate neutrophil function, and enhance immunity [32]. IL-12 is the strongest activator of NK cells. Strong hepatocyte immune responses and excessive hepatocyte apoptosis aggravate liver injury, and this aggravation is related to upregulation of IL-12 in CD8^+^ T cells [33]. TNF-*α* can induce the expression of inflammatory molecules by activating NF-*κ*B, which can aggravate liver tissue damage [34]. Binding of TNF-*α* to TNF-R1 on the hepatocyte membrane can break double-stranded genomic DNA into oligodeoxynucleotide fragments, which can increase the apoptosis of stem cells. IFN-*γ* can sensitize hepatocytes to TNF-*α*, making the hepatocytes more vulnerable to injury [35]. Oxidative stress following hepatic tissue damage has been found to increase the hepatic content of the inflammatory cytokines IFN-*γ*, IL-6, IL-12, and TNF-*α* [36]. In this study, Shoumei polyphenols alleviated hepatic injury by downregulating the serum levels of IFN-*γ*, IL-6, IL-12, and TNF-*α*, and their effects were comparable to those of silymarin.

nNOS preserves nerve cells, effectively protects tissue, and promotes the repair of damaged tissue [37]. The activity of eNOS in liver tissue is largely constant, and the NO generated by eNOS improves the healing of hepatic tissue. In addition, eNOS can promote liver vascular regeneration to repair damaged hepatic tissue [38]. When activated, iNOS can function for much longer than eNOS; thus, very large quantities of NO can be released. Moreover, low concentrations of NO can prevent gene mutation to a certain extent, which in turn improves physiological defence. However, excessive NO levels trigger gene mutation, cause pathological tissue changes, and increase the risks of genetic diseases [39]. Notably, oxidative stress can lead to overexpression of iNOS, nNOS, and eNOS, resulting in the exacerbation of hepatic injury [40]. In this study, Shoumei polyphenols reduced the expression of iNOS and elevated that of eNOS and nNOS to alleviate inflammatory responses, attenuate oxidative stress-related hepatic tissue damage, and protect against hepatic damage.

SOD is typically classified into two isomers, namely, Mn-SOD and Cu/Zn-SOD. Mn-SOD is a key isoenzyme that scavenges free radicals in the mitochondria, and its active center is Mn^4+^. Cu/Zn-SOD, the other isoenzyme, is involved in the scavenging of cytoplasmic free radicals, and its active center contains Cu^2+^ and Zn^2+^ [41]. It has been reported that Cu/Zn-SOD can abolish O_2_ toxicity and protect visceral tissue [42]. Following CCl_4_-induced hepatic injury, the activity of Mn-SOD decreases significantly [43]. Similar results were obtained in this study. Previous research has shown that CCl_4_ causes oxidative stress and the generation of massive amounts of free radicals; however, Cu/Zn-SOD and Mn-SOD can scavenge free radicals and prevent oxidative stress-induced hepatic injury [44]. Higher concentrations of H_2_O_2_ are associated with faster decomposition rates. CAT promotes the decomposition of H_2_O_2_ in hepatocytes and red blood cells and inhibits the production of toxic hydrogen and oxygen free radicals that harm the liver [45]. Our finvdings indicated that Shoumei polyphenols upregulated the hepatic expression of CAT, Cu/Zn-SOD, and Mn-SOD, thus ameliorating liver injury.

NF-*κ*B is an important transcription factor that can be activated by cytokines, chemokines, and/or growth factors [46]. In the inactive state, NF-*κ*B forms a complex with I*κ*B-*α* and then localizes to the cytoplasm. Upon inflammatory factor-induced NF-*κ*B stimulation, the I*κ*B-kinase complex is phosphorylated and activated by NF-*κ*B-inducible kinase, leading to I*κ*B-*α* phosphorylation at Ser32 and Ser36 and subsequent degradation by the ubiquitin-proteasome pathway [47]. After the depolymerization of NF-*κ*B and I*κ*B-*α*, the nuclear localization sequence is exposed, and NF-*κ*B is transported to the nucleus to promote NF-*κ*B-dependent gene transcription; this aggravates inflammation and eventually results in liver damage [48]. In this study, Shoumei polyphenols decreased NF-*κ*B mRNA levels and increased I*κ*B-*α* expression in cells with oxidative damage. Thus, Shoumei polyphenols regulated the expression levels of these two molecules *in vitro* and exerted antioxidant effects. The Shoumei polyphenols also reduced the mRNA and protein expression of NF-*κ*B p65 and enhanced that of I*κ*B-*α* in mice, thereby alleviating inflammation and inhibiting liver injury.

Polyphenol gallic acid has strong anti-free-radical and antioxidant effects and can be used to inhibit oxidative stress-induced DNA damage and protect liver tissue from free radicals [49]. Catechins have exhibited regulatory and antioxidant functions in cells, and their antioxidant effects are stronger than those of *α*-tocopherol. Catechins can clear free radicals produced in humans to strengthen cell membranes and have good biological activity [50]. Hyperoside also exhibits excellent antioxidant activity. Previous research has shown that hyperoside exerts an obvious protective effect on liver tissue and that its mechanism is related to its antioxidant activity, by which it promotes the return of NO to normal levels and improves SOD activity [51]. New research on sulfuretin has shown that this polyphenol can inhibit inflammation [52]. These four substances are responsible for the protective effects of Shoumei polyphenols against CCl_4_-induced liver injury. Two of these substances, hyperoside and sulfuretin, are not common in most teas, but their levels are high in white tea (Shoumei).

Shoumei tea is a kind of unfermented tea. Component analysis also found that it contains polyphenols which are different from those of ordinary green tea. Hyperoside and sulfuretin are very rare and are found in Shoumei tea, and both of these components show better antioxidant effects. Therefore Shoumei polyphenols are a special kind of tea polyphenols, which have better application value.

## 5. Conclusions

In this study, Shoumei polyphenols were found to protect the liver by regulating oxidation indexes *in vitro* and *in vivo*. Shoumei polyphenols had robust effects on CCl_4_-induced hepatic injury, and their activities were similar to those of silymarin. Shoumei polyphenols are found naturally in edible plants and exhibit high safety, no toxicity, and a lack of side effects; thus, they are valuable ameliorative agents. However, the possible synergistic effects of the key polyphenols in Shoumei remain to be further studied.

## Figures and Tables

**Table 1:** Gradient elution conditions of mobile phase.

Time (min)	Mobile phase A (%)	Mobile phase B (%)
0	88%	12%
15	80%	20%
35	60%	40%
50	30%	70%
55	0%	100%

**Table 2:** Sequences of primers used in the qPCR assay.

Gene name	Sequence
Bax (for human cell)	Forward: 5′-CCCGAGAGGTCTTTTTCCGAG-3′
	Reverse: 5′-CCAGCCCATGATGGTTCTGAT-3′
Bcl-2 (for human cell)	Forward: 5′-ATGTGTGTGGAGAGCGTCAACC-3′
	Reverse: 5′-CAGAGACAGCCAGGAGAAATCAA-3′
NF-*κ*B (for human cell)	Forward: 5′-GAAGCACGAATGACAGAGGC-3′
	Reverse: 5′-GCTTGGCGGATTAGCTCTTTT-3′
I*κ*B-*α* (for human cell)	Forward: 5′-GCTGAAGAAGGAGCGGCTACT-3′
	Reverse: 5′-TCGTACTCCTCGTCTTTCATGGA-3′
GAPDH (for human cell)	Forward: 5′-TCAAGAAGGTGGTGAAGCAGG-3′
	Reverse: 5′-AGCGTCAAAGGTGGAGGAGTG-3′
nNOS (for mice)	Forward: 5′-GAATACCAGCCTGATCCATGGAA-3′
	Reverse: 5′-TCCTCCAGGAGGGTGTCCACCGCATG-3′
eNOS (for mice)	Forward: 5′-TCAGCCATCACAGTGTTCCC-3′
	Reverse: 5′-ATAGCCCGCATAGCGTATCAG-3′
iNOS (for mice)	Forward: 5′-GTTCTCAGCCCAACAATACAAGA-3′
	Reverse: 5′-GTGGACGGGTCGATGTCAC-3′
Cu/Zn-SOD (for mice)	Forward: 5′-AACCAGTTGTGTTGTCAGGAC-3′
	Reverse: 5′-CCACCATGTTTCTTAGAGTGAGG-3′
Mn-SOD (for mice)	Forward: 5′-CAGACCTGCCTTACGACTATGG-3′
	Reverse: 5′-CTCGGTGGCGTTGAGATTGTT-3′
CAT (for mice)	Forward: 5′-GGAGGCGGGAACCCAATAG-3′
	Reverse: 5′-GTGTGCCATCTCGTCAGTGAA-3′
I*κ*B-*α* (for mice)	Forward: 5′-CGCGGGATGGCCTCAAGAAGGA-3′
	Reverse: 5′-GCCAAGTGCAGGAACGAGTCT-3′
NF-*κ*B (for mice)	Forward: 5′-GAGGCACGAGGCTCCTTTTCT-3′
	Reverse: 5′-GTAGCTGCATGGAGACTCGAACA-3′
GAPDH (for mice)	Forward: 5′-AGGTCGGTGTGAACGGATTTG-3′
	Reverse: 5′-GGGGTCGTTGATGGCAACA-3′

Bax: Bcl2-associated X protein; Bcl-2: B-cell lymphoma-2; NF-*κ*b: nuclear factor kappa B; I*κ*B-*α*: inhibitor of NF-*κ*B alpha; nNOS: neuronal nitric oxide synthase; eNOS: endothelial nitric oxide synthase; iNOS: inducible nitric oxide synthase; Mn-SOD: manganese superoxide dismutase; Cu/Zn-SOD: copper zinc-superoxide dismutase; CAT: catalase; GAPDH: glyceraldehyde-3-phosphate dehydrogenase.

**Table 3:** Linear regression equation for polyphenol compounds.

Types of compounds	Linear regression equation	*R* ^2^	Detection limit (ng/mL)	Precision RSD (%)	Repeatability RSD (%)	Content (mg/g)
Gallic acid	*y* = 13.133*x* − 7.4047	0.9983	0.25	0.12	0.68	90.555
Catechin	*y* = 8.5179*x* − 2.0695	0.9977	0.25	0.14	1.21	552.300
Hyperoside	*y* = 1.4453*x* + 0.3967	0.9935	0.30	0.20	0.87	47.565
Sulfuretin	*y* = 2.4597*x* + 0.1246	0.9992	0.25	0.16	0.44	19.980

**Table 4:** The liver index of mice treated with CCl_4_.

Group	Body weight (g)	Live weight (g)	Liver index
Normal	32.24 ± 1.12^a^	1.24 ± 0.06^d^	3.84 ± 0.22^d^
Model	32.23 ± 0.78^a^	2.91 ± 0.14^a^	9.03 ± 0.38^a^
Silymarin	32.14 ± 1.24^a^	1.66 ± 0.10^c^	5.16 ± 0.44^c^
SPL	32.04 ± 1.16^a^	2.59 ± 0.09^b^	8.09 ± 0.41^b^
SPH	31.82 ± 1.25^a^	1.79 ± 0.11^c^	5.61 ± 0.35^c^

Values presented are the mean ± standard deviation (*N* = 10/group). Sample data in each group come from normal distribution, and the difference in variance between the two groups was significant (*P* > 0.05). ^a-d^Mean values with different letters over the bar are significantly different (*P* < 0.05) according to Tukey's honestly significant difference. Normal: untreated mice; model: CCl_4_-treated mice; SPL: 100 mg/kg Shoumei polyphenols and CCl_4_-treated mice; SPH: 200 mg/kg Shoumei polyphenols and CCl_4_-treated mice; silymarin: 200 mg/kg silymarin and CCl_4_-treated mice.

**Table 5:** Levels of AST, ALT, ALP, TG, TC, BUN, and ALB in mice serum (*N* = 10).

Group	ALT (U/L)	AST (U/L)	ALP (K-A)	TG (mmol/L)	TC (mmol/L)	BUN (mg/dL)	ALB (g/dL)
Normal	19.04 ± 1.79^d^	10.77 ± 1.02^d^	5.83 ± 0.53^d^	0.34 ± 0.05^d^	1.31 ± 0.15^d^	19.01 ± 0.89^d^	4.15 ± 0.15^a^
Model	73.39 ± 2.82^a^	62.64 ± 2.40^a^	21.00 ± 1.27^a^	2.63 ± 0.15^a^	6.02 ± 0.18^a^	55.70 ± 2.75^a^	1.61 ± 0.09^e^
Silymarin	30.72 ± 1.84^c^	18.40 ± 1.26^c^	8.01 ± 0.43^c^	0.76 ± 0.08^c^	2.81 ± 0.11^c^	28.78 ± 0.93^c^	3.47 ± 0.11^b^
SPL	60.37 ± 2.85^b^	40.19 ± 2.06^b^	15.94 ± 1.05^b^	1.87 ± 0.12^b^	4.54 ± 0.20^b^	44.02 ± 2.08^b^	2.30 ± 0.10^d^
SPH	34.41 ± 2.35^c^	19.30 ± 1.14^c^	8.70 ± 0.38^c^	0.86 ± 0.06^c^	2.96 ± 0.10^c^	30.76 ± 1.40^c^	3.17 ± 0.08^c^

Values presented are the mean ± standard deviation (*N* = 10/group). Sample data in each group come from normal distribution, and the difference in variance between the two groups was significant (*P* > 0.05). ^a-d^Mean values with different letters over the bar are significantly different (*P* < 0.05) according to Tukey's honestly significant difference. Normal: untreated mice; model: CCl_4_-treated mice; SPL: 100 mg/kg Shoumei polyphenols and CCl_4_-treated mice; SPH: 200 mg/kg Shoumei polyphenols and CCl_4_-treated mice; silymarin: 200 mg/kg silymarin and CCl_4_-treated mice. AST: aspartate aminotransferase; ALT: alanine aminotransferase; ALP: alkaline phosphatase; TG: triglycerides; TC: total cholesterol; BUN: blood urea nitrogen; ALB: albumin.

**Table 6:** Levels of SOD, NO, CAT, MDA, and GSH-Px in serum of mouse (*N* = 10).

Group	SOD (U/mL)	NO (*μ*mol/L)	CAT (U/mL)	MDA (*μ*mol/L)	GSH-Px (U/mL)
Normal	140.38 ± 8.33^a^	47.26 ± 4.33^d^	42.68 ± 2.83^a^	5.43 ± 0.32^d^	266.38 ± 7.81^a^
Model	42.11 ± 4.99^d^	137.61 ± 5.86^a^	9.10 ± 1.26^d^	22.65 ± 1.34^a^	82.25 ± 4.70^d^
Silymarin	97.39 ± 5.33^b^	83.98 ± 3.28^c^	30.00 ± 1.17^b^	7.99 ± 0.14^c^	205.38 ± 12.45^b^
SPL	70.76 ± 6.01^c^	119.68 ± 6.10^b^	15.24 ± 0.97^c^	15.32 ± 0.76^b^	139.92 ± 8.11^c^
SPH	94.91 ± 3.01^b^	87.95 ± 4.39^c^	29.27 ± 1.10^b^	8.25 ± 0.18^c^	193.25 ± 12.21^b^

Values presented are the mean ± standard deviation (*N* = 10/group). Sample data in each group come from normal distribution, and the difference in variance between the two groups was significant (*P* > 0.05). ^a-d^Mean values with different letters over the bar are significantly different (*P* < 0.05) according to Tukey's honestly significant difference. Normal: untreated mice; model: CCl_4_-treated mice; SPL: 100 mg/kg Shoumei polyphenols and CCl_4_-treated mice; SPH: 200 mg/kg Shoumei polyphenols and CCl_4_-treated mice; silymarin: 200 mg/kg silymarin and CCl_4_-treated mice. SOD: superoxide dismutase; NO: nitric oxide; CAT: catalase; MDA: malondialdehyde; GSH-Px: glutathione peroxidase.

**Table 7:** Serum levels of IL-6, IL-12, TNF-*α*, and IFN-*γ* in mice (*N* = 10).

Group	IL-6 (pg/mL)	IL-12 (pg/mL)	TNF-*α* (pg/mL)	IFN-*γ* (pg/mL)
Normal	24.97 ± 1.41^d^	155.90 ± 8.07^d^	17.27 ± 1.59^d^	13.65 ± 1.01^d^
Model	209.69 ± 9.97^a^	822.17 ± 19.89^a^	132.95 ± 8.29^a^	84.49 ± 4.33^a^
Silymarin	82.20 ± 2.57^c^	352.09 ± 14.76^c^	45.67 ± 2.38^c^	37.11 ± 2.79^c^
SPL	137.59 ± 5.36^b^	627.25 ± 23.98^b^	96.97 ± 7.32^b^	60.58 ± 2.60^b^
SPH	86.56 ± 2.46^c^	369.57 ± 20.38^c^	48.15 ± 2.45^c^	42.71 ± 3.65^c^

Values presented are the mean ± standard deviation (*N* = 10/group). Sample data in each group come from normal distribution, and the difference in variance between the two groups was significant (*P* > 0.05). ^a-d^Mean values with different letters over the bar are significantly different (*P* < 0.05) according to Tukey's honestly significant difference. Normal: untreated mice; model: CCl_4_-treated mice; SPL: 100 mg/kg Shoumei polyphenols and CCl_4_-treated mice; SPH: 200 mg/kg Shoumei polyphenols and CCl_4_-treated mice; silymarin: 200 mg/kg silymarin and CCl_4_-treated mice. IL-6: interleukin 6; IL-12: interleukin 12; TNF-*α*: tumour necrosis factor alpha; IFN-*γ*: interferon gamma.

## Data Availability

No data were used to support this study.
